# Prospective randomized study comparing the Teleflex Medical SaphLITE Retractor to the Ethicon CardioVations Clearglide Endoscopic System

**DOI:** 10.1186/1749-8090-1-24

**Published:** 2006-09-06

**Authors:** Scot C Schultz, Dennis Stapleton, Paula D'Ambra, Cynthia Loftis, Christine Wahrmann, George Ebra

**Affiliations:** 1 Gulf Coast Cardiothoracic Surgeons, Naples Community Hospital, Naples, Florida, USA

## Abstract

**Background:**

Several minimally invasive saphenous vein harvesting techniques have been developed to reduce morbidities associated with coronary artery bypass grafting. This prospective, randomized study was designed to compare two commonly used minimally invasive saphenous vein harvesting techniques, the SaphLITE Retractor System (Teleflex Medical) and the Clearglide Endoscopic Vessel Harvesting System (Ethicon CardioVations, Inc.).

**Methods:**

Between January 2003 and March 2004, a total of 200 patients scheduled for primary, nonemergent coronary artery bypass grafting, with or without concomitant procedures were randomized into two groups: SaphLITE (n = 100) and Clearglide (n = 100). Pre-, intra- and postoperative data was collected and subjected to statistical analysis. Randomization provided homogenous groups with respect to preoperative risk factors.

**Results:**

Harvest location for the SaphLITE group was thigh (n = 40), lower leg (n = 5) and both lower leg and thigh (n = 55). The location of harvest for the Clearglide group was thigh (n = 3), lower leg (n = 16) and both lower leg and thigh (n = 81). The mean incision length was 3.6 cm (range, 2–6) in the SaphLITE group versus 2.1 cm (range, 1–4) in the Clearglide group (p < 0.05). The total incision length was 12.9 cm versus 8.9 (p < 0.05) in the SaphLITE and Clearglide groups. Conversion to the open technique occurred in 5 SaphLITE patients and 7 Clearglide patients. Intraoperative leg exploration for bleeding occurred in two of the Clearglide patients and none of the SaphLITE patients. Post-operative complications specifically related to minimally invasive harvesting technique, including a two-week post-discharge visit, were not statistically different between the groups.

**Conclusion:**

The saphenous vein can be safely harvested utilizing the SaphLITE and Clearglide systems. While the Clearglide system allows for fewer incisions (number and length) and less harvest time, these benefits may be outweighed by the increased cost of the Clearglide system compared to the SaphLITE retractor.

## Background

Coronary artery bypass grafting (CABG) is the most frequently performed cardiac surgical procedure in the world. There are approximately 467,000 CABG procedures performed in this country annually [1]. Along with the internal mammary and radial arteries, the greater saphenous vein (SV) is a commonly used conduit in CABG. Historically, the incision required for the removal of the SV has been 30–45 cm long and may at times extend from the thigh to the ankle. As a result, the harvest of the SV is commonly associated with increased morbidity including but not limited to pain, discomfort, scarring and the increased risk of wound infection. Wound morbidity rates of up to 24% have been reported with conventional (longitudinal) SV harvest techniques [2-9]. Occurrence of these events often negatively affects the patient's recovery phase and dominates the rehabilitative process [10].

During the past decade, several minimally invasive vein harvesting techniques have been developed to reduce morbidities associated with saphenous vein removal [11]. Minimally invasive procedures have demonstrated several advantages including reduced incidence of wound complications, decreased hospital length of stay, reduced scarring, etc. [12,13]. These potential benefits have prompted surgical groups to further explore minimally invasive harvest techniques (MIHT) and to discern their impact on health-care savings. In today's cost-containment environment, resource utilization becomes an important factor in the selection of an appropriate system.

The application of minimally invasive harvest is rapidly becoming the standard of care in most cardiac surgical practices. However, the efficacy, unresolved clinical concerns and cost benefits of various approaches have not been well-defined. This study examines clinical outcomes and discerns the cost benefits attributed to each of the technologies.

## Methods

### Study design

This was a prospective, randomized study comparing the use of two commonly-used MIHT. Random assignment was performed by means of drawing a sealed, unlabeled, unordered envelope from a container in the operating room. Patients were randomly assigned to one of two harvest techniques: the Teleflex Medical SaphLITE Retractor (SVH) or the Ethicon Cardiovations Clearglide Endoscopic Vessel Harvesting System (CVH). All patients in the study gave written, informed consent. This study was approved by the Institutional Review Board at Naples Community Hospital.

#### Inclusion criteria

Men and women 18 years of age or over undergoing elective or urgent CABG or CABG with a concomitant procedure, e.g., valve repair, valve replacement, etc were eligible for participation. Patients had to be willing and able to provide informed consent and to adhere to the required follow-up.

#### Exclusion criteria

Patients with morbid obesity (greater than 20% of ideal body weight), prior vein stripping and ligation, severe varicosities, preoperative infection or sepsis, evidence of leg ulcers and/or a surgical category of emergent or salvage were excluded from enrollment in the study. In addition, patients with previous CABG were also excluded.

### Patient population

From January 2003 to March 2004, 200 patients undergoing isolated CABG or CABG with a concomitant procedure, e.g., valve repair, valve replacement, etc. comprised the clinical material for this report. The SVH group was comprised of 81 men (81.0%) and 19 women (19.0%) with a mean age of 68.6 ± 9.4 years (range, 43–88 years). The CVH group included 80 men (80.0%) and 20 women (20.0%) with a mean age of 70.3 ± 9.4 years (range, 49–90 years). The clinical characteristics of the two patient groups are listed in Table 1. Based on the preoperative clinical characteristics measured, the study groups were comparable.

**Table 1:** Medical History Information by Patient Group*

Variables	SaphLITE (n = 100)	Clearglide (n = 100)	p Value
Smoking history	54 (54.0)	49 (49.0)	0.479
Chronic obstructive pulmonary disease	11 (11.0)	10 (10.0)	0.818
Diabetes mellitus	33 (33.0)	34 (34.0)	0.881
Renal insufficiency	6 (6.0)	2 (2.0)	0.149
Peripheral vascular disease	10 (10.0)	16 (16.0)	0.207
History of deep vein thrombosis	2 (2.0)	0 (0.0)	0.155
Varicose veins (current)	0 (0.0)	4 (4.0)	0.043
Steroids/Immunosuppressant Therapy	1 (1.0)	1 (1.0)	1.000
IIb/IIIa platelet inhibitors	7 (7.0)	2 (2.0)	0.088
Antiplatelet therapy	79 (79.0)	82 (82.0)	0.592
Antithrombotic therapy	35 (35.0)	24 (24.0)	0.088

* Data are expressed as No. (%).

### Definitions

#### Postoperative vein harvest site morbidities

*Erythema *referred to redness of the skin. *Ecchymosis *referred to a small hemorrhagic spot, larger than a petechia, in the skin or mucous membrane forming a nonelevated, rounded or irregular, blue or purplish patch. *Fluctuance *referred to a palpable indication of the presence of pus in a bacterial infection. *Hematoma *referred to a localized collection of blood. *Seroma *referred to a collection of serum in the tissues. *Wound dehiscence *referred to a superficial, partial or complete separation of the layers of a surgical wound. *Cellulitis *referred to an acute, diffuse, spreading, edematous, inflammation of deep subcutaneous tissues. *Blisters *referred to a vesicle having either watery or bloody contents. *Leg (harvest site) infection *referred to an infection involving a leg vein harvest site and which must have had either the wound opened with excision of tissue, positive culture or treatment with antibiotics.

#### Vein harvesting technique

All conduits are harvested by experienced physician assistants. The legs are prepped with chlorhexidine gluconate and betadine and draped with Ioban. The legs are then inspected for varicosities and a harvest site selected. A 2–3 cm incision is made below the knee medial to the tibia or above the knee at the medial inner thigh. Blunt dissection is performed to locate the greater SV.

#### Ethicon clearglide endoscopic approach

The Clearglide Optical Vessel Dissector was then placed over a 30 degree angle scope and attached to an endoscopic camera. The dissector allows for smooth atraumatic dissection on the anterior and lateral surfaces of the SV and creates a cavity for instrument passage. The dissector is then removed and the ultra-retractor is placed over the scope and into the tunnel. The retractor is then advanced until a side branch is identified. An Allport endoscopic clip applier is then used to clip the branch distally from the vein and endoscopic scissors are used to ligate the branch. This process is repeated distally and proximally through the tunnel as long as the length of the retractor allows until all side branches are secured. A metal vessel dissector hook is then used to assure that all side branches are ligated before transecting and removing the SV. A metal clip is then placed at the distal end of the vein allowing transaction and removal through the initial incision. A stab incision is then made proximally and the SV identified and secured with a 0-silk and/or metal clip, transected and finally removed.

#### Teleflex medical SaphLITE approach

The SaphLITE self-retaining system is attached to the operative bed to allow hands-free retraction and visualization. A disposable light panel is fit into a blade, which is attached to the SaphLITE retractor. The retractor is then inserted into the tunnel and gently advanced for direct visualization of the SV. A metal hook dissector and scissors are used to dissect the SV and to identify side branches. Once identified, branches are clipped with metal clips and ligated. Once the end of the blade is reached, another incision is made if additional vein length is required. Blunt dissection is again used to identify the SV and to create a tunnel. This process is continued proximally and distally until the desired length of SV is obtained. The harvested vein is then ligated at both ends with a 0-silk and/or metal clip, transected and removed.

Following the application of either harvesting technique, the vein is prepared to be used as a conduit for CABG. A plastic cannula is inserted into the distal end of the SV and a solution of albumin, heparin and saline is used to flush out the SV. Side branches are tied with 3-0 silk and/or clipped with metal clips.

### Operative data

The left leg was the harvest site for 97 patients (97.0%) in each group. Five patients (5.0%) were converted to an open approach in the SVH group and 7 patients (7.0%) in the CVH group. The SV quality was ideal in 51 patients (51.0%) in the SVH group and in 37 patients (37.0%) in the CVH group. Moreover, the SV quality was found to be acceptable in 44 patients (44.0%) in the SVH group and in 55 (55.0%) in the CVH group. In 5 patients (5.0%) in the SVH group and 8 patients (8.0%) in the CVH group, the SV was of poor quality. There was no re-exploration for bleeding in the SVH group; however, 2 patients (2.0%) in the CVH group had excessive bleeding and required re-exploration. Information on selected intraoperative variables is presented in Table 2.

**Table 2:** Vein Harvest Intraoperative Variables by Patient Group*

Variables	SaphLITE (n = 100)	Clearglide (n = 100)	p Value
Number of incisions performed	3.6 ± 1.1	2.1 ± 0.8	0.001
Total incision length (cm)	12.9 ± 5.6	9.0 ± 9.1	0.001
Length of saphenous vein harvested (cm)	38.5 ± 12.9	43.5 ± 13.6	0.010
Total saphenous vein harvest time (min)	47.6 ± 17.2	41.8 ± 16.8	0.014
Number of saphenous veins repaired	0.8 ± 1.1	1.1 ± 1.2	0.084

* Values given as mean ± SD.

In the SVH group, the number of incisions performed and the total incision length was significantly higher (p = 0.001) than in the CVH group. Moreover, in the CVH group the length of the SV harvested was significantly greater (p = 0.010) than in the SVH group. The total SV harvest time was significantly greater (p = 0.014) in the SVH group than in the CVH group. The SV was removed at a rate of 0.80 cm/minute with the SVH compared to 1.04 cm/minute with the CVH. Harvest with the SVH required fewer repairs; however this difference did not achieve statistical significance.

### Data collection and management

Preoperative, intraoperative, postoperative and follow-up data collection was accomplished for each patient participating in the study. Data were obtained by prospective review of the patient's hospital record, catheterization reports, echocardiography and pathology reports applying a standardized methodology and definition of terms. The principle research technique involved the completion of a *Saphenous Vein Harvest Study-Hospital Case Report Form *during the patient's hospitalization. Moreover, a *Follow-up Case Report Form *was completed during the patient's office follow-up visit at 2 weeks post-discharge. A *Telephone Follow-up Report Form *was completed with information collected through a telephone interview conducted at 12 weeks post-discharge from the hospital.

All follow-up information was obtained through direct patient contact, family members or the patient's personal physician. The use of these data collection instruments provided for standardized reporting of each patient's clinical status before and after the operation. Follow-up was 98.0% complete in the SVH group with 2 patients being lost to follow-up after discharge from the hospital. In the CVH group, follow-up was 100.0% complete with no patients lost after discharge from the hospital. Data collected were entered into a Microsoft Excel spreadsheet (Microsoft Inc.) and subsequently retrieved for analysis.

### Statistical methods

Data are presented as frequency distributions and simple percentages. Values of continuous variables are expressed as mean ± standard deviation. Univariate analysis of selected preoperative and postoperative discrete variables was accomplished by chi-square, the continuity-adjusted chi-square analysis or a two-tailed Fisher's exact test with the appropriate degrees of freedom to test for the equality of proportions in the case of categorical variables. Comparison of means for continuous variables was conducted by an unpaired Student's *t*-test. A significant difference between measurements was defined as *p *less than or equal to 0.05. Data collected were subjected to both quantitative and qualitative analysis using the *Number Cruncher Statistical Systems *(*NCSS*), Kaysville, UT.

## Results

### In-hospital events

The hospital mortality rate for the SVH group was 3.0% (3/100) and 2.0% (2/100) for the CVH group. The overall hospital mortality rate for the series was 2.5% (5/200). The hospital morbidities relating to the SV harvest site for the two groups are presented in Table 3. There were no significant differences in the occurrence rate of postoperative harvest site morbidities between the groups. Except for the occurrence of ecchymosis, the overall rate of occurrences of harvest site complications was low for both groups.

**Table 3:** Leg Wound Appearance Following Hospital Discharge by Patient Group*

	In-Hospital			Office Follow-up		
Variables	SaphLITE (n = 100)	Clearglide (n = 100)	p Value	SaphLITE (n = 95)	Clearglide (n = 96)	p Value
Erythema	5 (5.0)	2 (2.0)	0.243	7 (7.4)	10 (10.4)	0.459
Echymosis	19 (19.0)	20 (20.0)	0.886	8 (8.4)	8 (8.3)	0.983
Hematoma	2 (2.0)	1 (1.0)	0.555	3 (3.2)	1 (1.0)	0.307
Seroma	2 (2.0)	2 (2.0)	0.992	2 (2.1)	4 (4.2)	0.414
Wound dehiscence	0 (0.0)	0 (0.0)	--	1 (1.1)	1 (1.0)	0.994
Cellulitis	3 (3.0)	0 (0.0)	0.079	1 (1.1)	3 (3.1)	0.317
Wound drainage	4 (4.0)	1 (1.0)	0.171	2 (2.1)	4 (4.2)	0.414
Blisters	0 (0.0)	1 (1.0)	0.319	1 (1.1)	0 (0.0)	0.314
Leg (harvest site) infection	0 (0.0)	0 (0.0)	--	0 (0.0)	1 (1.0)	0.319

* Data are expressed as No. (%).

The assessment of leg edema on a scale of 0 to 4, with 0 representing no edema and 4 representing severe edema, was similar for the two groups. In the SVH group 84 patients (86.1%) and 83 patients (84.7%) in the CVH group had no edema (mean, 0.144 versus 0.214 respectively). Information concerning the patient's ability to ambulate following surgery revealed similar outcomes. Fifty-three patients (54.6%) in the SVH group and 56 patients (57.1%) in the CVH group were able to ambulate on the first postoperative day (mean, 1.9 days versus 2.0 days respectively).

At hospital discharge, the patient's leg pain was assessed on a scale of 0 to 10 with 0 representing no pain and 10 the worst pain. In the SVH group, 70 patients (72.0%) and 69 patients (70.0%) in the CVH group reported no pain (mean, 0.526 versus 0.592 respectively). The mean postoperative length of stay for patients in the SVH group was 6.8 ± 5.0 days and 7.3 ± 4.4 days for the CVH group. This difference did not achieve statistical significance.

#### Patient follow-up

Office follow-up was conducted at 2 weeks post-discharge from the hospital. Two patients (2.1%) in the SVH group and 7 patients (7.3%) in the CVH group required antibiotic therapy. Seventy-six patients (80.0%) in the SVH group and 81 patients (84.4%) in the CVH group were completely healed when seen at office follow-up. Leg wound appearance was assessed at the office follow-up by patient group (see Table 3). No significant difference was noted in the various factors assessed between the groups in leg wound appearance.

The assessment of leg edema at office follow-up was conducted using a scale of 0 to 4, with 0 representing no edema and 4 representing severe edema. In the SVH group, 76 patients (80.0%) and 70 patients (72.9%) in the CVH group had no edema (mean, 0.231 versus 0.365 respectively). An assessment of leg pain was performed at office follow-up, utilizing a scale of 0 to 10 with 0 representing no pain and 10 the worst pain. In the SVH group, 69 patients (72.6%) and in the CVH group 71 patients (74.0%) reported no pain (mean, 0.526 versus 0.592 respectively). Ambulation assessment at 2 weeks post discharge revealed that 59 patients (62.1%) in the SVH group and 58 patients (60.4%) were walking more than a half-mile per day.

At 12 weeks following discharge from the hospital, a telephone interview was conducted with each patient. There were 3 patients (3.2%) in the SVH group and 2 patients (2.6%) in the CVH group who were receiving antibiotic therapy for leg related problems. Moreover, only 1 patient (1.1%) in the SVH group and no patients in the CVH group underwent incision and drainage for seroma.

Leg pain assessment at telephone follow-up found 77 patients (81.1%) in the SVH group and 77 patients (78.6%) in the CVH group experiencing no pain (0.368 versus 0.398) respectively. There were 71 patients (74.7%) in the SVH group and 66 patients (67.3%) in the CVH group who were walking more than a half-mile per day. No patient in either group required a hospital readmission for leg wound complications.

### Cost-benefit analysis

The capital cost for the purchase of the SVH system is approximately $19,235 whereas the cost of the CVH system is $30,000. This includes the 30 degree videoscope and video equipment. In addition, a monitor system is required for the CVH system which adds an additional $2,500 to the overall cost of the system. As for the cost per case, the SVH system requires a light panel which is $210 versus $509 for the CVH system which includes a vessel dissector, ultra retractor, endoscopic clip applier and endoscopic scissors. Excluding the cost of capital equipment, the disposable supplies for the present study was $21,000 for the SVH system versus $50,900 for the CVH system. This results in a cost savings of $29,100 for the 200 patients participating in the present study and demonstrates a financial benefit for the use of the SVH system when compared to the CVH approach.

## Discussion

During the past decade, major advances have been made in the application of minimally invasive technology in cardiovascular surgery. Minimally invasive SV harvesting has become a standard of care at most institutions. It is associated with diminished morbidity, decreased postoperative pain, improved cosmesis and reduced the rate of readmission for leg wound complications [14-16]. As a result, health care costs may be reduced and patient's satisfaction and quality of life should be improved.

These benefits to the patient and the health care organization must be considered against the increased costs associated with MIHT. Moreover, these initial benefits of the procedure must be considered in concert with long-term graft patency. Some investigators have expressed concern regarding vein integrity with the use of less invasive methods of SV harvesting [12,17]. However, Fabricius et al [18] and Griffith et al [19] have documented minimal morphologic differences in histology when comparing the application of minimally invasive techniques in SV harvest with the traditional open approach.

This study has clearly demonstrated the benefits, safety and efficacy of SV harvesting utilizing the SVH or the CVH system. It must be recognized that these approaches to SV harvesting are technically demanding and involve a learning curve. To avoid any concern regarding the technical competence of the harvesters (physician assistants) in the present study, they were required to perform 50 cases with each system prior to the conduct of the study.

The conversion to an open approach was low in the present study, with 5 patients (5.0%) in the SVH group and 7 patients (7.0%) in the CVH group converted to an open approach. These results are comparable to those reported by Crouch et al [20] who documented a conversion rate of 7.2% (13/180) and Allen et al [12] who indicated a 5.6% rate (3/54). Conversion to an open approach often occurs when visualization through the camera becomes difficult as a result of bleeding. Moreover, conversions may be a function of patients having minimal subcutaneous tissue or those with fragile veins. Our protocol requires that the procedure be converted to an open technique any time the SV being harvested is compromised or at risk for injury.

Significant differences were noted between the groups based on intraoperative variables. The SVH group had a greater number of incisions than the CVH group (p = 0.001). This is to be expected as the CVH system has a greater length than the SVH. Moreover, the total incision length was greater for the SVH group than the CVH group (p = 0.001). The length of SV harvested was greater with the CVH techniques and the time to perform the harvest was also shorter (p = 0.014). A greater number of saphenous veins were repaired in the CVH group (1.1 ± 1.2) than in the SVH group (0.8 ± 1.1). However, this difference did not achieve statistical significance. The number of SV repairs in the present series was lower than that previously reported [21].

Early postoperative (in-hospital) leg wound appearance demonstrated no significant difference between the groups. At office follow-up, again no significant difference in leg wound appearance was noted in the two groups. Similar results were also reported at telephone follow-up. No patient in either group required a hospital readmission for leg wound complications. These outcomes are more favorable than those reported in similar studies of minimally invasive SV harvesting [22-24]. The assessment of leg wound pain between the groups revealed no significant difference with the majority of patients reporting no pain. Similar findings were documented at telephone follow-up. Minimal to non-existent perceived leg pain has been previously reported using minimally invasive SV harvesting systems [25,26].

The SVH harvest technique was more time consuming than the CVH approach (p = 0.014). However the time difference for the two approaches was approximately 6 minutes and did not delay, in any manner, the surgical procedure. As a result, harvest time should not be a factor in selecting an appropriate SV harvesting system.

In today's cost containment health care environment, the SVH approach offers an attractive alternative when compared to the increased per-case cost of the CVH system. However, the CVH system allows for fewer incisions (number and length) and less harvest time. Based on these findings, it may be that the use of these two systems should be individualized based on the patient's requirements.

With the SVH system, there is a tendency to harvest thigh vein, whereas there is a tendency to use the CVH system for the lower leg. In cases where it is documented or suspected that severe peripheral vascular disease exists below the knee and one to two segments of SV are needed, the SVH system appears to be the approach of choice. Furthermore, if only one segment of SV is required, the SVH system should be considered for harvesting above the knee. On the other hand, if an entire leg of SV vein is required, the application of CVH technology is the application of choice. The surgeon must weight the available options and select the system based on the individualized needs of the patient.

## Conclusion

Minimally invasive SV harvesting is a safe and effective technique to secure the required conduit for CABG. This approach increases patient satisfaction, enhances quality of life and provides for less pain and scarring to the individual.

This study has demonstrated that the two minimally invasive methods of SV harvesting have comparable outcomes. However, the CVH system has an added cost that must be factored into the selection algorithm in today's cost-conscious health care environment.

## Abbreviations

CABG = coronary artery bypass grafting

MIHT = minimally invasive harvesting techniques

SV = saphenous vein

SVH = SaphLITE

CVH = Clearglide
